# Role of raphe magnus 5-HT_1A_ receptor in increased ventilatory responses induced by intermittent hypoxia in rats

**DOI:** 10.1186/s12931-022-01970-6

**Published:** 2022-03-03

**Authors:** Jiao Su, Yang Meng, Yifei Fang, Linge Sun, Mengge Wang, Yanjun Liu, Chunling Zhao, Liping Dai, Songyun Ouyang

**Affiliations:** 1 Department of Respiratory and Sleep Medicine, First Affiliated Hospital of Zhengzhou University, No. 1 Jianshe East Road, Erqi District, Zhengzhou, 450052 Henan China; 2 Henan Institute of Medical and Pharmaceutical Sciences, Zhengzhou University, Henan, 450052 China

**Keywords:** Intermittent hypoxia, Ventilation, Raphe magnus nucleus, 5-HT_1A_ receptor

## Abstract

**Background:**

Intermittent hypoxia induces increased ventilatory responses in a 5-HT-dependent manner. This study aimed to explore that effect of raphe magnus serotonin 1A receptor (5-HT_1A_) receptor on the increased ventilatory responses induced by intermittent hypoxia.

**Methods:**

Stereotaxic surgery was performed in adult male rats, and acute and chronic intermittent hypoxia models were established after recovery from surgery. The experimental group received microinjections of 5-HT_1A_ receptor agonist 8-hydroxy-2-(di-n-propylamino)tetralin (8-OH-DPAT) into the raphe magnus nucleus (RMg). Meanwhile, the control group received microinjections of artificial cerebrospinal fluid instead of 8-OH-DPAT. Ventilatory responses were compared among the different groups of oxygen status. 5-HT expressions in the RMg region were assessed by immunohistochemistry after chronic intermittent hypoxia.

**Results:**

Compared with the normoxia group, the acute intermittent hypoxia group exhibited higher ventilatory responses (e.g., shorter inspiratory time and higher tidal volume, frequency of breathing, minute ventilation, and mean inspiratory flow) (P < 0.05). 8-OH-DPAT microinjection partly weakened these changes in the acute intermittent hypoxia group. Further, compared with the acute intermittent hypoxia group, rats in chronic intermittent hypoxia group exhibited higher measures of ventilatory responses after 1 day of intermittent hypoxia (P < 0.05). These effects peaked after 3 days of intermittent hypoxia treatment and then decreased gradually. Moreover, these changes were diminished in the experimental group. 5-HT expression in the RMg region increased after chronic intermittent hypoxia, which was consistent with the changing trend of ventilatory responses. While activation of the 5-HT_1A_ receptor in the RMg region alleviated this phenomenon.

**Conclusions:**

The results indicate that RMg 5-HT_1A_ receptor, via changing the expression level of 5-HT in the RMg region, is involved in the modulation of the increased ventilatory responses induced by intermittent hypoxia.

**Supplementary Information:**

The online version contains supplementary material available at 10.1186/s12931-022-01970-6.

## Background

Hypoxia is generally divided into sustained hypoxia and intermittent hypoxia according to its pattern. It has been suggested that sustained hypoxia is defined as an uninterrupted period of hypoxia [1, 2], whereas intermittent hypoxia is defined as hypoxia with a hiatus, and the period of the interval in which does not exist hypoxia lasts from a few seconds to several hours [3, 4]. Different patterns of hypoxia lead to different ventilatory responses. Intermittent hypoxia, the unique characteristic of sleep apnea, is caused by repeated upper airway collapse during sleep in patient with obstructive sleep apnea (OSA) [5], and is generally divided into acute intermittent hypoxia (AIH) and chronic intermittent hypoxia (CIH) according to its cumulative duration. Previous studies have suggested that the cumulative duration of AIH should not exceed 1 day and ranges from several minutes to 8 h [6–8], whereas CIH is performed on consecutive days, namely, the cumulative duration of CIH lasts more than 1 day [8–10]. AIH could induce increased ventilatory responses in rats [7] and humans [11], which can be enhanced by CIH, but cannot be elicited by sustained hypoxia [12, 13]. The increased ventilatory responses induced by intermittent hypoxia is hypothesized to be a physiological compensation for promoting breathing stability in conditions that otherwise lead to apnea or hypopnea during sleep in patients with OSA. Determining the mechanism by which intermittent hypoxia induces increased ventilatory response in rats has important implications for understanding compensation in patients with OSA and guiding neuropharmacological interventions to ensure normal respiratory function during sleep.

Changes in 5-HT system are thought to be associated with the increased ventilatory responses in mammals [14]. There is an anatomical support for the interaction between raphe magnus (RMg) 5-HT neurons and the nuclei involved in respiratory control in the brainstem, including hypoglossal nuclei [15], phrenic motor nucleus [16], and ventral respiratory column [17–20]. And electrical stimulation of the RMg region played an inhibitory role on the excitability of phrenic motoneurones and medullary respiratory neurones [21–23]. Furthermore, RMg is one of the midline brainstem cell groups thought to be involved in physiological responses to hypoxia [7, 11, 24]. We previously found that CIH can increase genioglossus activity that can be suppressed by the specific lesioning of RMg 5-HT neurons [25]. The 5-HT_1_ receptor family is distributed in different regions of the brain and brainstem in mammals, with 5-HT_1_ receptors widely distributed in the RMg region [26, 27]. 5-HT_1A_ receptors are divided into various receptor types according to anatomical location; these include autoreceptor of 5-HT neurons, heteroreceptor of non-5-HT neurons and extra-synaptically [28]. The 5-HT_1A_ receptor in the RMg 5-HT neurons is a typical presynaptic autoreceptor [29] that can inhibit the release of presynaptic neurotransmitters and information transmission between synapses [30].

Dodig and his colleagues have found that AIH-induced phrenic long-term facilitation (LTF) was inhibited by microinjection of WAY-100635 into the caudal raphe region [31]. While the caudal raphe region mainly includes the raphe obscurus nucleus, raphe pallidus nucleus, RMg, and parapyramidal region. Previous studies discovered that 5-HT_1A_ receptor in the RMg region modulate the ventilatory responses under another form of hypoxia-sustained hypoxia [32] and hypercapnia [33]. Different patterns of hypoxia lead to different ventilatory responses. Nevertheless, it remains unknown whether IH-induced increased ventilatory responses are modulated by 5-HT_1A_ receptor in the RMg 5-HT neurons. Thus, the present study aimed to explore the influence of 5-HT_1A_ receptor in the RMg 5-HT neurons in ventilatory responses during AIH and CIH in rats.

## Methods

### Animals

Specific pathogen-free adult male Sprague–Dawley rats weighing 250–300 g were purchased from Liaoning Changsheng Biotechnology Co., Ltd. Rats were provided free access to drinking water and food. The feeding conditions were as follows: temperature at 24 ± 2 ℃ and relative air humidity at 40%, and the illumination cycle was as follows: lights on at 8:00 and lights off at 20:00. All procedures were approved by the ethics committee of First Affiliated Hospital of Zhengzhou University and were performed in accordance with the National Institute of Health Guide for Care and Use of Laboratory Animals. All efforts were made to relieve rats’ suffering and reduce the number of rats used.

### Surgery

Rats were anesthetized with chloral hydrate and then placed in a stereotaxic apparatus (68003, RWD Life Science, China). Stereotactic surgery was performed as described in our previous study [25]. The coordinates of RMg were 10.52–11.30 mm from the bregma in the midline and 10.4 mm below the dorsal surface of the skull according to the Paxinos and Watson atlas [34]. The procedure for guide cannula and the matched injection cannula implantation into the RMg region was described in previous studies [35]. Solutions were microinjected through the injection cannula at a fixed rate of 0.05 mL/h [33].

### Intermittent hypoxia

AIH and CIH treatments were both performed 1 week postoperatively. AIH treatment was performed in the subject chamber of whole-body plethysmograph. The subject chamber was flushed with a mixture of gas (N_2_, 88%; O_2_, 12%) for 5 min and air for 5 min, for 10 cycles in total. Rats in the CIH group were placed in commercial chambers (Oxycycler model A48XOV; BioSherix, NY) with hypoxia (10% O_2_, 45 s) and normoxia (21% O_2_, 60 s) every 188 s, 8 h/d (8:00–16:00), for 4 weeks as described in our previous study [25]. Rats in the corresponding control group were subjected to identical experimental conditions under air in parallel. Agitation was observed in all rats for no more than 5 min at the 1st day of CIH. In addition, there were no significant difference in body weight of rats in each group during the 4-week experiment.

### Drugs

OH-DPAT (Sigma) is a highly selective 5-HT_1A_ receptor agonist that can specifically bind to 5-HT_1A_ receptors in the presynaptic membrane and suppress the excitability of RMg 5-HT neurons. Previous studies have confirmed that 0.01–30 mM 8-OH-DPAT can inhibit the excitability of 5-HT neurons [33, 36–38], while 10 mM and 30 mM 8-OH-DPAT had the side effect of blood pressure fluctuations, which made the animal fidget, in addition to the above effects [33]. Therefore, we selected an 8-OH-DPAT concentration of 1 mM in the present study. Artificial cerebrospinal fluid (ACSF) [25] was injected instead of 8-OH-DPAT in the control group.

### Whole-body plethysmography

Ventilatory responses were measured noninvasively using whole-body barometric plethysmograph (EMKA, France), as described in a previous study [39]. The plethysmograph consists of two chambers, that is, subject chamber (4300 mL) and reference chamber (2000 mL). Freely behaving rats were placed in the subject chamber, which was connected to the reference chamber by a controlled leak. The atmosphere in the subject chamber was maintained with dry air at a speed of 2 L/min. A differential transducer measured the pressure difference between the two chambers. Ventilatory measures were derived from this measured pressure signal. The acquired signals were analyzed with the analyzer of respiratory flow, which produced the parameters of inspiratory time (TI), expiratory time (TE), frequency of breathing (F), tidal volume (VT), minute ventilation (VE), mean inspiratory flow (VT/TI), peak inspiratory flow, end-inspiratory pause, peak expiratory flow, end-expiratory pause, expired volume, relaxation time, enhanced pause and relative humidity, temperature, and atmospheric pressure in the subject chamber.

### Experimental protocol

#### Protocol 1. Effects of RMg 5-HT_1A_ receptor on ventilatory responses in AIH rats

This protocol was performed in 36 rats. Rats were randomly divided into six groups: the AIH (n = 6), AIH + ACSF (n = 6), AIH + 8-OH-DPAT (n = 6) and the corresponding control groups, the Control (n = 6), Control + ACSF (n = 6), Control + 8-OH-DPAT (n = 6) groups.

Rats in the AIH + 8-OH-DPAT, AIH + ACSF, Control + 8-OH-DPAT and Control + ACSF groups underwent the stereotactic surgery. After 1-week recovery, the rats in all group were placed into a plethysmograph at least 30 min prior to the start of ventilatory measurement. After rats had acclimated and appeared calm, the subject chambers in the AIH, AIH + ACSF, and AIH + 8-OH-DPAT groups were flushed with a gas mixture (N_2_, 88%; O_2_, 12%) for 5 min followed by air for another 5 min, for a total of 10 cycles. Rats in the Control, Control + ACSF and Control + 8-OH-DPAT groups were subjected to identical experimental conditions under air in parallel. Rats in the AIH + 8-OH-DPAT and Control + 8-OH-DPAT groups were microinjected with 8-OH-DPAT in the RMg region before the measurement. Rats in the AIH + ACSF and Control + ACSF groups were microinjected with ACSF instead. The ventilatory responses were measured continuously for 60 min.

#### Protocol 2. Effects of 4-week CIH on ventilatory responses in rats

This protocol was performed on 12 rats equally divided into two groups as the CIH group (n = 6) and the normoxia (NO) group (n = 6). The CIH group received CIH treatment, whereas the NO group was subjected to identical experimental conditions under air in parallel. Ventilatory responses were measured before CIH and after 1 day, 3 days, 1 week, 2 weeks, 3 weeks, and 4 weeks of CIH treatment.

#### Protocol 3. Effects of RMg 5-HT_1A_ receptor on ventilatory responses in CIH rats

This protocol was performed in 40 rats. Rats were divided into four groups: the CIH + 8-OH-DPAT (n = 10), CIH + ACSF (n = 10), NO + 8-OH-DPAT (n = 10), and NO + CSF (n = 10) groups. Stereotactic surgery was performed in all rats. At 1 week postoperatively, the CIH + 8-OH-DPAT and CIH + ACSF groups were subjected to CIH for 4 consecutive weeks. Meanwhile, the NO + 8-OH-DPAT and NO + ACSF groups were subjected to air condition. All groups were placed into the plethysmograph at least 30 min prior to the start of ventilatory measurement.

After rats had acclimated and appeared calm, the solution was microdialysed. The CIH + 8-OH-DPAT and NO + 8-OH-DPAT groups received 8-OH-DPAT microinjections into the RMg region. Meanwhile, the NO + ACSF and CIH + ACSF groups were microinjected with ACSF. Ventilatory responses were measured before CIH and after 1 day, 3 days, 1 week, 2 weeks, 3 weeks, and 4 weeks of CIH treatment.

#### Protocol 4. Effect of 5-HT_1A_ receptor on the expression of 5-HT in raphe magnus nucleus in CIH rats

Rats were divided into six groups according to stimulus (CIH) and treatment (drug). The CIH, CIH + 8-OH-DPAT, CIH + ACSF groups; and the corresponding control groups (the Air, Air + 8-OH-DPAT and Air + ACSF groups). Rats in the control groups were subjected to identical experimental conditions under air in parallel. The rats in each group were further divided into six subgroups according to the total duration of CIH. Rats in the CIH group were subdivided into the CIH 1 day, CIH 3 days, CIH 1 week, CIH 2 weeks, CIH 3 weeks, CIH 4 weeks subgroups (*n* = 6 in each group). Rats in the CIH + 8-OH-DPAT group were subdivided the CIH + 8-OH-DPAT 1 day, CIH + 8-OH-DPAT 3 days, CIH + 8-OH-DPAT 1 week, CIH + 8-OH-DPAT 2 weeks, CIH + 8-OH-DPAT 3 weeks, CIH + 8-OH-DPAT 4 weeks subgroups (*n* = 6 in each group). Rats in the corresponding control group (Air, Air + 8-OH-DPAT, Air + ACSF and CIH + ACSF groups) were also divided into six subgroups to exclude the influence of age, surgery and solvent of 8-OH-DPAT.

After the above experiment, rats were immediately anesthetized by intraperitoneal injection of 10% chloral hydrate, then intracardially perfused with a phosphate-buffered solution (PBS), and fixed by 4% paraformaldehyde. After fixation, the brainstem was paraffin, and then the RMg region were cut into 5-µm coronal Sects. six sections per rat used for microinjection site verification and six sections per rat for 5-HT immunohistochemistry.

### Microinjection site verification

Rats were anesthetized and fixed as described above. After fixation, Evan’s blue (60 nL, Sigma) was microinjected in RMg region. The brainstem was paraffin and then cut into 5 micron coronal sections. Finally, the sections were stained with neutral red for site verification. Data from rats with correct stereoscopic site of RMg region were included in data analysis.

### 5-HT immunohistochemistry

Sections were baked at 60ºC for 3 h, dewaxed, hydrated progressively, and then the endogenous peroxidase was inactivated with 3% H_2_O_2_ for 10 min. After washing in PBS, antigens were retrieved by citric acid buffer (0.01 M, PH 6.0) microwave antigen retrieval. The sections were incubated in normal non-immune goat serum at room temperature for 50 min, followed by incubation with polyclonal rabbit anti-5-HT antibody (1:800, ab10385, Abcam) overnight at 4 °C. After washing in PBS, the sections were incubated with biotinylated goat anti-rabbit secondary antibody for 60 min, horseradish enzyme-labelled streptomycin ovalbumin for 60 min at room temperature, and DAB kit (Beijing Zhongshan Jinqiao Biotechnology Co., LTD, Beijin, China) for 10 min in succession. Finally, the slides were progressively dehydrated with alcohol, transparent with xylene, and closed with cover-slips.

Negative controls were set up as blank samples. PBS was used instead of the primary antibodies, and the staining results were negative. These results indicated that the method was reliable and could exclude endogenous tissue peroxidase, alkaline phosphatase, spontaneous fluorescence, and other substances that could cause nonspecific colour rendering.

The 5-HT-immunoreactive (5-HT-ir) cells in the RMg region sections were imaged using a computerised system that included a microscope (BX51, Olympus, Japan) and a charge-coupled device micrographic system (U-CMAD3, Olympus, Japan). The membrane and/or cytoplasm of the 5-HT-ir cells in the RMg region are indicated in brown; the cell bodies are round or oval with protuberances, appearing as brown circles under high-power magnification. The 5-HT expression level was described as mean optical density (MOD) using Image-Pro 6.0 (Media Cybemetics, INC., Rockville, MD, USA). Higher MODs indicated higher protein expression.

### Statistical analyses

Data are reported as the means ± standard deviation. The measurement of ventilatory responses was collected at 15-min intervals using the following formula: VE (mL/[min × 100 g]) = (VE_15min_ + VE_30min_ + VE_45min_ + VE_60min_)/4. Measures were rejected from the analysis when the rats were moving or asleep.

One-way analysis of variance (ANOVA) was used to evaluate ventilatory measures among the AIH and corresponding control groups. Ventilatory measures among the CIH and corresponding control groups were analyzed using a repeated measures multivariate ANOVA, with three factors: treatment (drug), time (day), and stimulus (CIH). One-way ANOVA was used to analyze significance interactions. The Duncan test was performed for multiple comparisons. One-way analysis of variance compared the 5-HT expression level among the groups. LSD t-test were used to analyze significance interactions if the test of homogeneity of variance is uniform, otherwise, Dunnett's T test was used. All statistical analyses were performed using the Statistical Package for the Social Sciences version 16.0 for Windows. P < 0.05 was considered statistically significant.

## Results

### Effects of RMg 5-HT_1A_ receptor on ventilatory responses in AIH rats

**Fig. 1:**
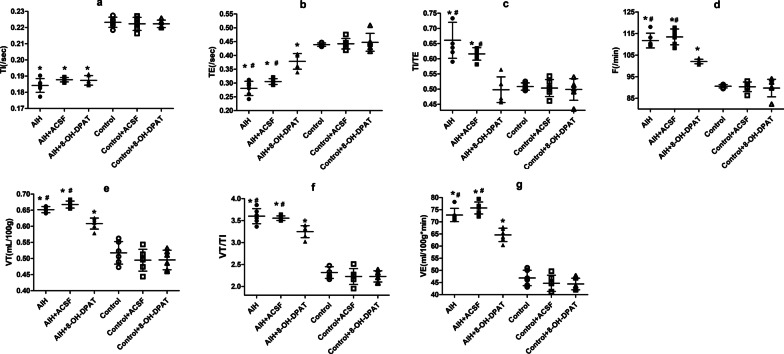
Effect of RMg 5-HT_1A_ receptor on ventilatory responses after AIH. **a** Represents the comparisons of TI between the AIH, AIH + ACSF, AIH + 8-OH-DPAT groups and the corresponding control groups; **b** The comparisons of TE; **c** The comparisons of TI/TE; **d** The comparisons of F, **e** The comparisons of VT, **f** The comparisons of VT/TI, and **g** The comparisons of VE. *Indicates a significant difference between the AIH, AIH + ACSF, AIH + 8-OH-DPAT groups and the corresponding control group (*P* < 0.05). #Indicates a significant difference when compared with AIH + 8-OH-DPAT group (*P* < 0.05)

Six rats in each group were included in the analysis. There were no significant differences in the ventilatory responses (TI, TE, TI/TE, F, VT, VT/TI and VE) between the Control and Control + ACSF groups (Fig. 1, *P* > 0.05). Similar results were obtained between the AIH and AIH + ACSF groups (Fig. 1, *P* > 0.05). Stereotaxic surgery and ACSF microinjection had no significant effect on ventilatory responses. Ventilatory responses (TI, TE, TI/TE, F, VT, VT/TI and VE) were also no significant differences between the Control, Control + ACSF groups and the Control + 8-OH-DPAT group (Fig. 1, *P* > 0.05). It indicated that the 5-HT_1A_ receptor in the RMg region had no significant effect on ventilatory responses under normoxia.

Compared with the Control group, TI was significantly shortened in the AIH (shortened by 17.51%, Fig. 1a; *P* < 0.05) and AIH + 8-OH-DPAT groups (shortened by 15.90%, Fig. 1a; *P* < 0.05), but there was no significant difference between the AIH and AIH + 8-OH-DPAT groups; TI/TE was increased significantly in the AIH group (increased by 29.94%, Fig. 1c; *P* < 0.05), while there was no significant difference between the Control and AIH + 8-OH-DPAT groups (0.5086 ± 0.0119 vs. 0.4979 ± 0.0422, Fig. 1c; *P* > 0.05); TE was shortened, and F, VT, VT/TI and VE were increased in the AIH and AIH + 8-OH-DPAT groups, while the extent of change were all significantly weaker in the AIH + 8-OH-DPAT group (Control vs. AIH vs. AIH + 8-OH-DPAT group, TE: 100% vs. 63.90% vs. 86.15%; Fig. 1b; F: 100% vs. 123.32% vs. 112.61%; Fig. 1d; VT: 100% vs. 125.88% vs. 117.54%, Fig. 1e; VT/TI: 100% vs. 155.37% vs. 140.22%, Fig. 1f; VE: 100% vs. 155.32% vs. 137.83%, Fig. 1g; all *P* < 0.05). These data indicated that AIH could increase the ventilatory capacity (VT, VE, and F) and ventilatory drive (VT/TI) in adult rats, while activation of the 5-HT_1A_ receptors in the RMg region alleviated this phenomenon.

### Effects of CIH on ventilatory responses in rats

**Fig. 2:**
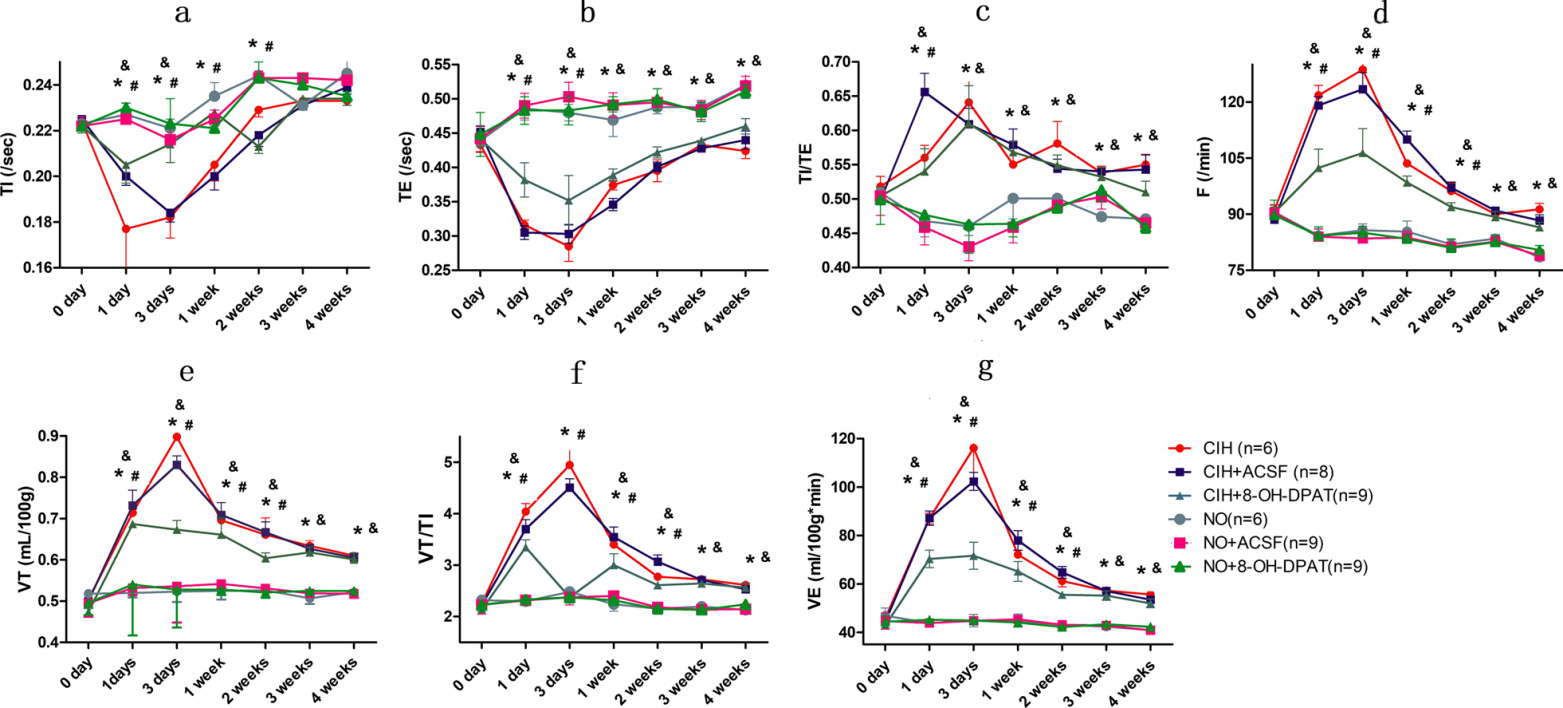
Effect of RMg 5-HT_1A_ receptor on ventilatory responses after CIH. **a** Represents the comparisons of TI after 1-day, 3-day, 1-week, 2-week, 3-week and 4-week CIH treatment between the CIH, CIH + ACSF, CIH + 8-OH-DPAT groups and the corresponding control groups; **b** the comparisons of TE; **c** the comparisons of TI/TE; **d** the comparisons of F, **e** the comparisons of VT, **f** the comparisons of VT/TI, and **g** the comparisons of VE. *Indicates *P* < 0.05 between the CIH, CIH + ASCF groups and the NO, NO + ACSF groups at the same measurement time; &Indicates *P* < 0.05 between the CIH + 8-OH-DPAT group and the NO + 8-OH-DPAT group at the same measurement time; **#**Indicates *P* < 0.05 between the CIH, CIH + ACSF groups and the CIH + 8-OH-DPAT group at the same measurement time

Six rats in the CIH and NO groups were included in the analysis. TI and TE were shortened, and TI/TE, F, VT, VT/TI and VE were increased after 1-day CIH treatment in the CIH group compared with the baseline (TI: shortened by 20.98%, Fig. 2a; TE: shortened by 27.02%, Fig. 2b; TI/TE: increased by 8.11%, Fig. 2c; F: increased by 33.71%, Fig. 2d; VT: increased by 41.67%, Fig. 2e; VT/TI: increased by 79.37%, Fig. 2f; VE: increased by 89.46%, Fig. 2g; all *P* < 0.05), and compared with those in the NO group at the same measurement time (TI: shortened by 22.03%, Fig. 2a; TE: shortened by 34.98%, Fig. 2b; TI/TE: increased by 19.66%, Fig. 2c; F: increased by 44.51%, Fig. 2d; VT: increased by 37.31%, Fig. 2e; VT/TI: increased by 75.78%, Fig. 2f; VE: increased by 98.49%, Fig. 2g; all *P* < 0.05); and these effects, except for TI (vs. baseline, shortened by 18.75%; vs. NO group, shortened by 17.65%, Fig. 2a), peaked after 3-day CIH treatment (vs. baseline, TE: shortened by 34.18%, Fig. 2b; TI/TE: increased by 23.75%, Fig. 2c; F: increased by 41.27%, Fig. 2d; VT: increased by 78.17%, Fig. 2e; VT/TI: increased by 119.61%, Fig. 2f; VE: increased by 152.96%, Fig. 2g; vs. NO group, TE: shortened by 40.63%, Fig. 2b; TI/TE: increased by 39.35%, Fig. 2c; F: increased by 50.07%, Fig. 2d; VT: increased by 71.70%, Fig. 2e; VT/TI: increased by 99.36%, Fig. 2f; VE: increased by 159.00%, Fig. 2g; *P* < 0.05) and then decreased gradually. These results indicated that CIH could increase the ventilatory responses, whereas this effect was weakened beyond a certain extent and duration.

### Effects of RMg 5-HT_1A_ receptors on ventilatory responses in CIH rats

8 rats in the CIH + ACSF group, 9 rats in the CIH + 8-OH-DPAT, NO + 8-OH-DPAT and NO + 8-OH-DPAT groups were included in the analysis. Compared with the NO group, the NO + ACSF and NO + 8-OH-DPAT groups had no significant difference in the ventilatory responses (TI, TE, TI/TE, F, VT, VT/TI and VE) (Fig. 2; *P* > 0.05). Similar results were obtained between the CIH and CIH + ACSF groups (*P* > 0.05, Fig. 2). It suggested that stereotaxic surgery and ACSF microinjection had no significant effect on ventilatory responses in CIH rats.

TI and TE were both shortened, and TI/TE, F, VT, VT/TI and VE were all increased after 1-day CIH treatment in the CIH + 8-OH-DPAT group compared with baseline (TI: shortened by 7.66%, Fig. 2a; TE: shortened by 13.38%, Fig. 2b; TI/TE: increased by 7.14%, Fig. 2c; F: increased by 13.20%, Fig. 2d; VT: increased by 44.94%, Fig. 2e; VT/TI: increased by 57.04%, Fig. 2f; VE: increased by 64.17%, Fig. 2g; all P < 0.05), and compared with those in the NO + 8-OH-DPAT group at the same measurement time (TI: shortened by 10.87%, Fig. 2a; TE: shortened by 20.91%, Fig. 2b; TI/TE: increased by 13.21%, Fig. 2c; F: increased by 21.88%, Fig. 2d; VT: increased by 29.14%, Fig. 2e; VT/TI: increased by 44.31%, Fig. 2f; VE: increased by 60.46%, Fig. 2g; all *P* < 0.05); while the extent of these changes in ventilatory responses were significantly weaker in the CIH + 8-OH-DPAT group than those in the CIH group (Fig. 2, *P* < 0.05). These data indicated that the increased ventilatory responses induced by CIH could be suppressed by activation of 5-HT_1A_ receptors in the RMg region.

### Differences in ventilatory responses in the AIH + 8-OH-DPAT group and the CIH + 8-OH-DPAT group

Compared with AIH group, the AIH + 8-OH-DPAT group had no significantly difference in TI (0.1842 ± 0.0043 s vs. 0.1874 ± 0.0029 s, Fig. 1a; *P* > 0.05); while compared with CIH group, TI was prolonged by 16.92% after 1-day CIH treatment in the CIH + 8-OH-DPAT group (0.1769 ± 0.0057 s vs. 0.2055 ± 0.0079 s, Fig. 2a; *P* < 0.05). Compared with the AIH group, the AIH + 8-OH-DPAT group were decreased by 8.68% in F, 6.63% in VT, 9.75% VT/TI and 11.26% in VE (Fig. 2, *P* < 0.05). As the ventilatory responses (TE, TE/TI, F, VT, VT/TI and VE) peaked after 3-day CIH treatment, the comparison were carried out between the CIH and CIH + 8-OH-DPAT groups after 3-day CIH treatment, and found that F was decreased by 17.31%, VT decreased by 25.04%, VT/TI decreased by 52.04%, and VE decreased by 38.33% in CIH + 8-OH-DPAT group than those in the CIH group (Fig. 2, *P* < 0.05). It indicated that daily repeated administration of 8-OH-DPAT have a stronger inhibitory effect on ventilatory responses in rats with CIH than those in rats with AIH.

### Effect of 5-HT_1A_ receptor on the expression of 5-HT in raphe magnus nucleus during chronic intermittent hypoxia

**Fig. 3:**
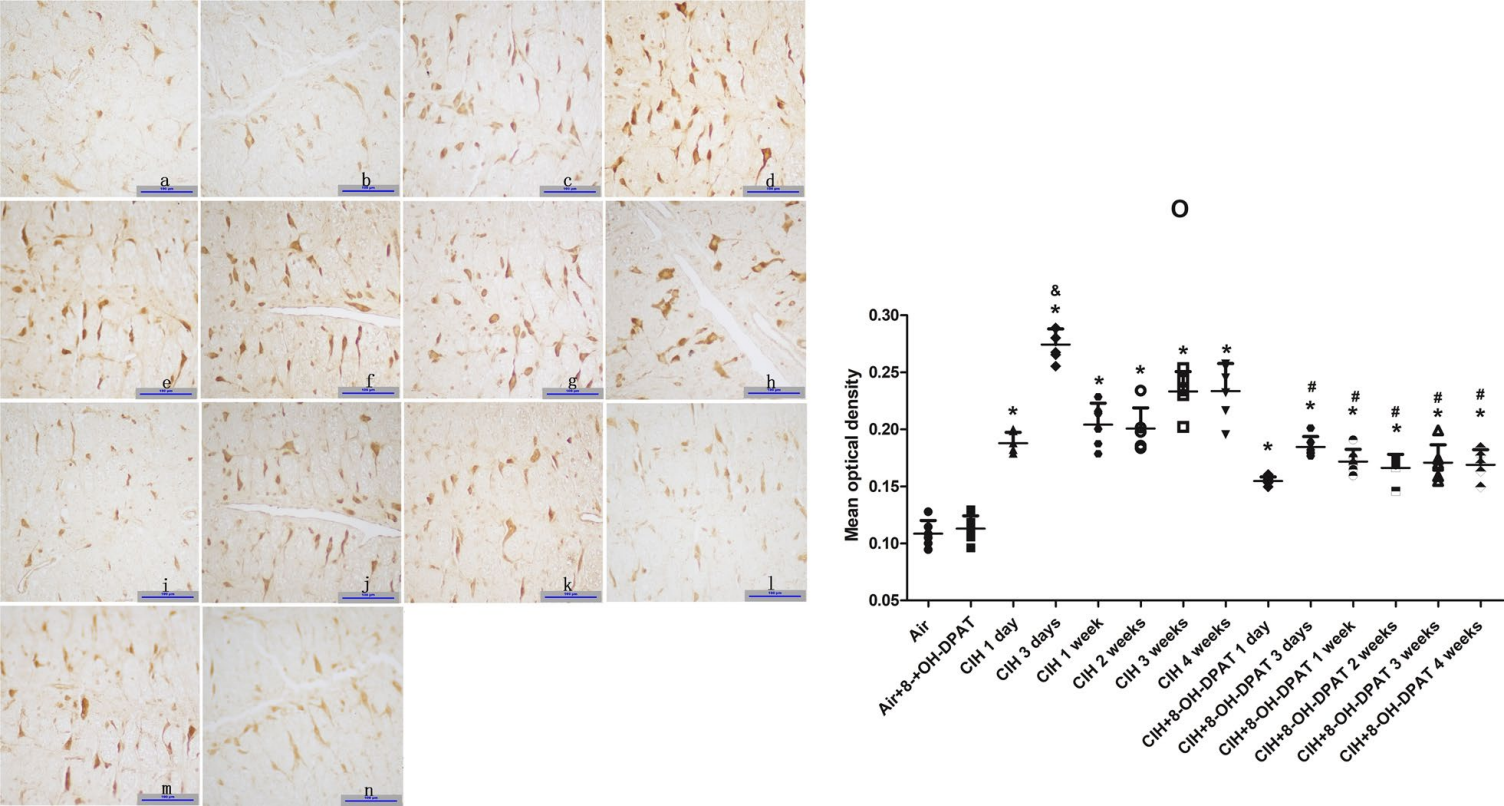
Comparisons of 5-HT expression in RMg region among the groups. **a** Represents a typical example of 5-HT-immunoreactive cells in the RMg region in the Air group; **b** the Air + 8-OH-DPAT group; **c** the CIH 1 day group; **d** the CIH 3 days group; **e** the CIH 1 week group; **f** the CIH 2 weeks group; **g** the CIH 3 weeks group; **h** the CIH 4 weeks group; **i** the CIH 1 day + 8-OH-DPAT group; **j** the CIH 3 days + 8-OH-DPAT group; **k** the CIH 1 week + 8-OH-DPAT group; **l** the CIH 2 weeks + 8-OH-DPAT group; **m** the CIH 3 weeks + 8-OH-DPAT group; **n** the CIH 4 weeks + 8-OH-DPAT group; **o** the comparisons of 5-HT expression in RMg region among the groups. *Indicates a significant difference compared to the Air group (*P* < 0.05), #indicates a significant difference compared to the corresponding CIH groups (*P* < 0.05), &indicates a significant difference compared to the CIH 1 day, CIH 1 week, CIH 2 weeks, CIH 3 weeks and CIH 4 weeks groups (*P* < 0.05)

6 rats in each group, except the CIH + 8-OH-DPAT 2 weeks (n = 5) and CIH + 8-OH-DPAT 4 weeks (n = 5) groups, were included in the analysis. The MOD of 5-HT was no significant difference in the Air, Air + 8-OH-DPAT and Air + ACSF groups, and results were similar in the CIH and CIH + ACSF groups (Fig. 3, *P* > 0.05). Changes in 5-HT expression in the RMg region are shown in Fig. 3.

The MOD of 5-HT in the RMg region of the CIH, CIH + 8-OH-DPAT groups were higher than those of the corresponding control groups (CIH vs. Air; CIH + 8-OH-DPAT vs. Air + 8-OH-DPAT; Fig. 3; *P* < 0.05). The MOD of 5-HT in the RMg region further increased and peaked after 3-day CIH treatment and gradually decreased thereafter in the CIH group (Fig. 3; *P* < 0.05). This was consistent with the changing trend of ventilatory responses after CIH. Compared with the CIH group, the MOD of 5-HT in the RMg region was lower in CIH + 8-OH-DPAT group (Fig. 3; *P* < 0.05); while there was no change trend similar to that in the CIH group.

## Discussion

It is unclear whether the IH-induced increased ventilatory response is mediated by the 5-HT_1A_ receptor in the RMg region. There were two major findings from this study. First, AIH induced an increased ventilatory response in rats, and CIH caused a more significant ventilatory response than that in rats with AIH, whereas this effect was weakened beyond a certain extent and duration. This phenomenon is referred to as ventilatory LTF in rats [7] and has also been observed in awake patients with OSA [11]. We further found that the increased ventilatory responses peaked after 3 days of CIH treatment and then weakened gradually. Second, the 5-HT_1A_ receptor in the RMg region, via changing the expression level of 5-HT in the RMg region, suppressed the increased ventilatory responses during AIH and CIH, other than normoxia. A previous study has found that AIH-induced phrenic LTF was inhibited by 5-HT_1A_ receptor antagonist in the caudal raphe region [31]. In this study, we used a more accurate nuclear localization (RMg, one of the raphe nuclei in the caudal raphe region) and a CIH pattern to investigate the effect of RMg 5-HT_1A_ receptor on the increased ventilatory responses induced by 4-week CIH.

In the present study, both measures of ventilatory capacity (VT, VE, and F) and ventilatory drive (VT/TI) were increased in AIH rats, and these increases were more pronounced in CIH rats than those in the sham controls. This phenomenon may be a manifestation of the compensatory ability of the ventilatory response induced by IH. Reeves et al. found that although ventilatory plasticity is age-dependent, it can be induced by CIH [40]. McGuire et al. have subjected rats to 1-week CIH and found that ventilatory LTF is time dependent, but it reached its peak after 8-h IH treatment [8]. Although slightly different, our findings are consistent with these previous results. The minor differences may be related to the variations in IH exposure paradigm [41], the substrain [42], and age of rats [40].

Ling et al. suggested that the CIH-induced ventilatory plasticity was 5-HT-dependent [43]. A previous study has also found that RMg 5-HT neurons are involved in the regulation of ventilatory response under sustained hypoxia [24]. We previously found that RMg 5-HT neurons modulated genioglossus corticomotor activity in CIH rats [25]. In the study by Pavlinac et al., 5-HT_1A_ receptor antagonist was microinjected intravenously, and they found that phrenic LTF can be inhibited by the 5-HT_1A_ receptor antagonist in anesthetized rats [44]. Nucci TB et al. have also found that the 5-HT_1A_ receptor antagonist decreased the ventilatory response in rats under sustained hypoxia [32]. Taylor et al. have demonstrated that 8-OH-DPAT increased the ventilatory response in rats under hypercapnia [33]. The present study used 8-OH-DPAT to investigate the effect of the 5-HT_1A_ receptor in the RMg region on ventilatory responses during AIH and CIH treatment in rats. The results showed that 8-OH-DPAT can suppress the effect of IH on ventilatory responses. Collectively, these findings indicate that the RMg 5-HT_1A_ receptor is involved in regulating the increased ventilatory responses during IH in rats.

Specific injury of medullary 5-HT neurons was reported to decrease the respiratory response to CIH in rats [25, 45]. Previous studies have shown that 5-HT expression was decreased significantly by sustained hypoxia in the dorsal raphe nucleus and RMg region [46, 47], while the 5-HT expression in hypoglossal nucleus was increased during CIH [48]. However, changes in the expression of 5-HT in the RMg region during CIH have not been reported. Our study discovered that 5-HT expression in the RMg region increased after CIH, and this was consistent with the changing trend of ventilatory responses after CIH. While activation of the 5-HT_1A_ receptor inhibited the 5-HT expression after CIH, and there was no change trend similar to that in the CIH group. This change is in contrast to the change in 5-HT expression caused by sustained hypoxia, which may explain why CIH but not sustained hypoxia, could induce the increased ventilatory responses.

This study has the following limitations. First, ventilatory responses were measured using a whole-body plethysmograph that monitored all the parameters as a whole. Activities of the upper airway, diaphragm, and intercostal muscle could not be measured separately. However, whole-body plethysmograph can detect the ventilatory responses in awake free-moving rats, avoid the effects of traumatic tracheotomy and anesthesia, and improve the convenience of performing the experimental procedures. Thus, it is suitable for long-term follow-up studies. Second, only male adult rats were included in the experiment; thus, we were unable to explore the influence of sex and age. The incidence of OSA differs by sex, and the increased ventilatory responses induced by IH are age-dependent. Thus, the influence of sex and age on ventilatory responses was not analyzed in the present study. Third, although ventilatory plasticity is related to the sleep–wake cycle and activity of rats, we only conducted whole-body plethysmography when the rats were quiet. However, this also eliminated the confounding factors (Additional file 1, Additional file 2).

## Conclusions

The results indicate that RMg 5-HT_1A_ receptor, via changing the expression level of 5-HT in the RMg region, is involved in the modulation of the increased ventilatory responses induced by IH. Further studies will focus on the mechanism of RMg 5-HT_1A_ receptor on the increased ventilatory responses induced by IH for guiding neuropharmacological interventions to modify ventilatory function during sleep in patients with OSA.

## Supplementary Information

**Additional file 1.** The typical respiratory waveforms under different conditions. **a**, rats under normoxia, **b**, rats after AIH. **c**, rats after 1 day’s IH.

**Additional file 2.** Representative 5-HT-ir cells in the RMg region. 5-HT-ir: 5-HT-immunoreactive; bas: basilar artery; ml: medial lemniscus; py:pyramidal tract; RPa: raphe pallidus nucleus; tth; trigemino thalamic tract.

## Data Availability

The datasets used and/or analysed during the current study are available from the corresponding author on reasonable request.

## Abbreviations

5-HT-ir cell: 5-HT-immunoreactive cell
ACSF: Artificial cerebrospinal fluid
AIH: Acute intermittent hypoxia
ANOVA: Analysis of variance
CIH: Chronic intermittent hypoxia
F: Frequency of breathing
IH: Intermittent hypoxia
MOD: Mean optical density
OSA: Obstructive sleep apnea
PBS: Phosphate-buffered solution
RMg: Raphe magnus nucleus
TE: Expiratory time
TI: Inspiratory time
VE: Minute ventilation
LTF: Long-term facilitation
VT: Tidal volume
