# Supplementary figures and images for: Role of raphe magnus 5-HT1A receptor in increased ventilatory responses induced by intermittent hypoxia in rats
Source: Respir Res. 2022 Mar 3;23:42. doi: 10.1186/s12931-022-01970-6 (PMC8892800; doi:10.1186/s12931-022-01970-6)

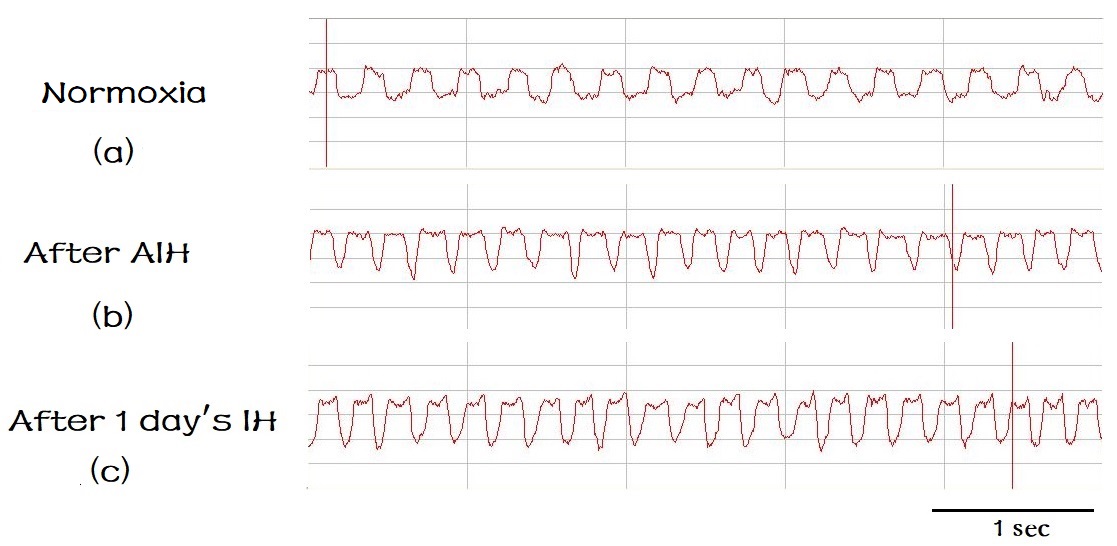

Supplement: Supplementary file 1 — Additional file 1. The typical respiratory waveforms under different conditions. a, rats under normoxia, b, rats after AIH. c, rats after 1 day’s IH. [file 12931_2022_1970_MOESM1_ESM.jpg]

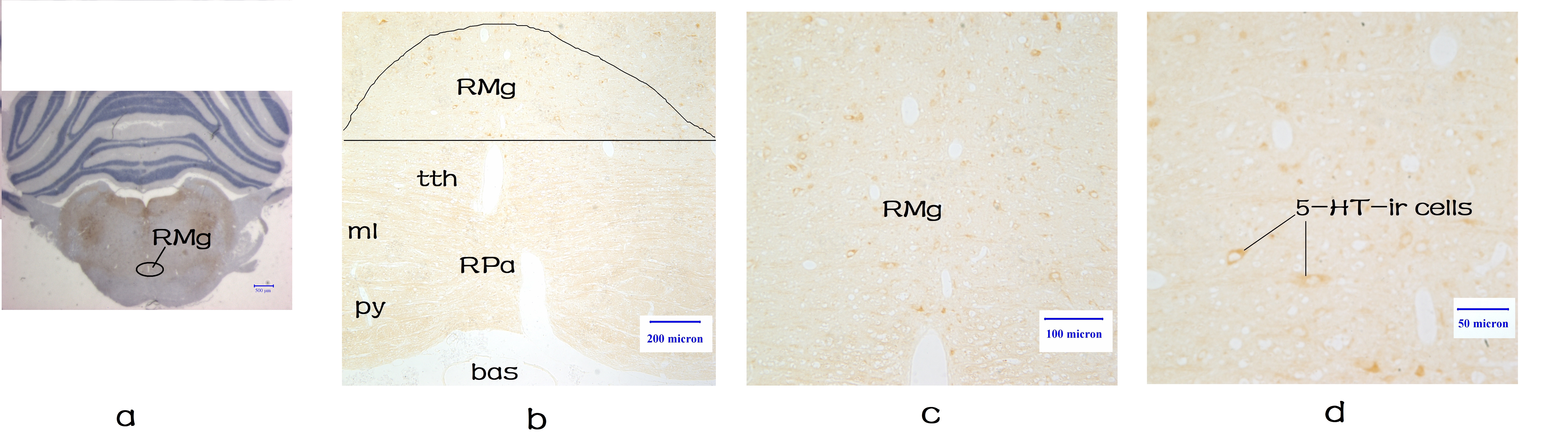

Supplement: Supplementary file 2 — Additional file 2. Representative 5-HT-ir cells in the RMg region. 5-HT-ir: 5-HT-immunoreactive; bas: basilar artery; ml: medial lemniscus; py:pyramidal tract; RPa: raphe pallidus nucleus; tth; trigemino thalamic tract. [file 12931_2022_1970_MOESM2_ESM.jpg]
